# Evaluation of the changes of orbital cavity volume and shape after tooth-borne and bone-borne rapid maxillary expansion (RME)

**DOI:** 10.1186/s13005-020-00235-1

**Published:** 2020-09-08

**Authors:** Antonino Lo Giudice, Lorenzo Rustico, Vincenzo Ronsivalle, Carmelo Nicotra, Manuel Lagravère, Cristina Grippaudo

**Affiliations:** 1 Department of Medical-Surgical Specialties – Section of Orthodontics, School of Dentistry, University of Catania, Policlinico Universitario “V. Emanuele,”, Via Santa Sofia 78, 95123 Catania, Italy; 2 DDS, MSc, Private Practice, Ispica (RG), Italy; 3 Orthodontic Graduate Program, University of Alberta, Edmonton, Alberta Canada; 4 Dental and Maxillofacial Institute, Head and Neck Department, Fondazione Policlinico Gemelli IRCCS, Catholic University of Sacred Heart, 00168 Rome, Italy

## Abstract

**Objective:**

To assess and compare volumetric and shape changes of the orbital cavity in patients treated with tooth-borne (TB) and bone-borne (BB) rapid maxillary expansion (RME).

**Study design:**

Forty adolescents with bilateral maxillary cross-bite received tooth-borne (TB group = 20**; mean age 14.27 ± 1.36 years**) or bone-borne (BB group = 20; mean age of 14.62 ± 1.45 years) maxillary expander. Cone-beam computed tomography (CBCT) were taken before treatment (T1) and 6-month after the expander activation (T2). Volumetric and shape changes of orbital cavities were detected by referring to a specific 3D digital technology involving deviation analysis of T1/T2 CBCT-derived models of pulp chamber. Student’s t tests were used to 1) compare T1 and T2 volumes of orbital cavities in TB and BB groups, 2) compare volumetric changes and the percentage of matching of 3D orbital models (T1-T2) between the two groups.

**Results:**

Both TB and BB groups showed a slight increase of the orbital volume (0.64 cm^3^ and 0.77 cm^3^) (*p* < 0.0001). This increment were significant between the two groups (*p* < 0.05) while no differences were found in the percentage of matching of T1/T2 orbital 3D models (*p* > 0.05). The areas of greater changes were detected in the proximity of the frontozygomatic and frontomaxillary sutures.

**Conclusion:**

TB-RME and BB-RME would not seem to considerably affect the anatomy or the volume of the orbital cavity in adolescents.

## Introduction

Rapid Maxillary expansion (RME) is the most efficient treatment for the correction of maxillary transverse deficiency [1]. Findings from the highest levels of evidence confirm that this protocol accomplishes transverse expansion of maxillary arch through skeletal and a dentoalveolar effects [1–3]. **However, previous studies based on finite element analyses (FEM) showed that RME increases the stress levels on neighboring structures and on the cranial base due to the cumulative forces released during the repeated activations of the expansion screw**[4, 5]**. It would seem that this cumulative force pattern can be responsible for the widening of the spheno-occipital synchondrosis in young subjects underwent RME** [6]**. Concerning the circummaxillary structures, post-treatment changes were found at the level of the zygomatic process, the frontozygomatic suture and the frontal process of the maxilla** [1, 7–9], **all these structures being part of the orbital cavity. In this regard,** previous evidence suggested that maxillary expansion may alter the volume of the orbital cavity [10], however this aspect has not been **studied extensively nor it has been determined if volumetric changes of the orbital cavity caused by RME may interfere with its normal growth pattern and physiology. This aspect could be of clinical relevance** considering that a positive correlation was found between the increment of orbital volumes and the degree of dysmorphology, enophtalmos, orbitopathy [11].

Skeletal anchorage has been proposed to increase the skeletal effect of RME, limiting dento-alveolar effects and widening the spectrum of patients to late adolescent and adults [12–14]. In this regard, bone-borne RME (or miniscrew-assisted RME) has showed greater skeletal effect compared to the tooth-borne RME [14–16]. As consequence, it could be postulated that the greater effectiveness of **miniscrew-**assisted RME could be associated with a greater effect on surrounding structures, such as the orbital cavity. Considering the prevalence of transverse maxillary deficiency among children and adolescent [17], awareness of the risks associated with the use of a tooth-borne and a bone-borne RME appears clinically relevant.

Nowadays, progress in radiographic techniques and 3D imaging software allows a more accurate evaluation and comparison of the morphological changes of anatomical structure. In particular, specific anatomical structures can be reconstructed (3D rendering) from CBCT scans, and superimposed to accurately evaluate growth or treatment changes [18]. Meanwhile, deviation analysis can be used to detect shape differences between two CBCT-derived anatomical models as well as to obtain precise dimensional information [19–21].

In this respect, the aim of the present study was to assess volumetric as well as morphological surface changes of the orbital cavity in patients treated with both tooth-borne and bone-borne rapid maxillary expansion, by referring to the surface-to-surface technique and deviation analysis of CBCT-derived 3D rendered models of the orbital cavity. **The null hypothesis was the absence of significant changes in the orbital cavity architecture between the two different protocols of skeletal maxillary expansion.**

## Materials and methods

The study obtained the approval of the Institutional Review Board of Indiana University–Purdue University (IRB protocol number: Pro00075765) and included a sample of adolescents with a diagnosis of skeletal transverse deficiency and who completed the orthodontic treatment at the Orthodontic Clinic of the University of Alberta (Edmonton, Canada, USA). **The study sample was obtained from previously published materials** [22], **in order to avoid unnecessary or additional radiation exposure to the patients.** All subjects were diagnosed with a bilateral maxillary cross-bite involving **at least the two maxillary first molars** and were treated with a tooth-borne (TB group) or a bone-borne (BB group) RME as part of their comprehensive orthodontic therapy. **Eligibility criteria** were as follow: 1) age between 11 and 15 years, 2) CBCT scans of good quality taken prior to the placement of the maxillary expander (T1) and after its removal (T2), 3) no artifact due to restorative materials present, 4) no previous orthodontic treatment, 5) no systemic disease or usage of medication, 6) no craniofacial syndromes or anomalies.

**Fig. 1:**
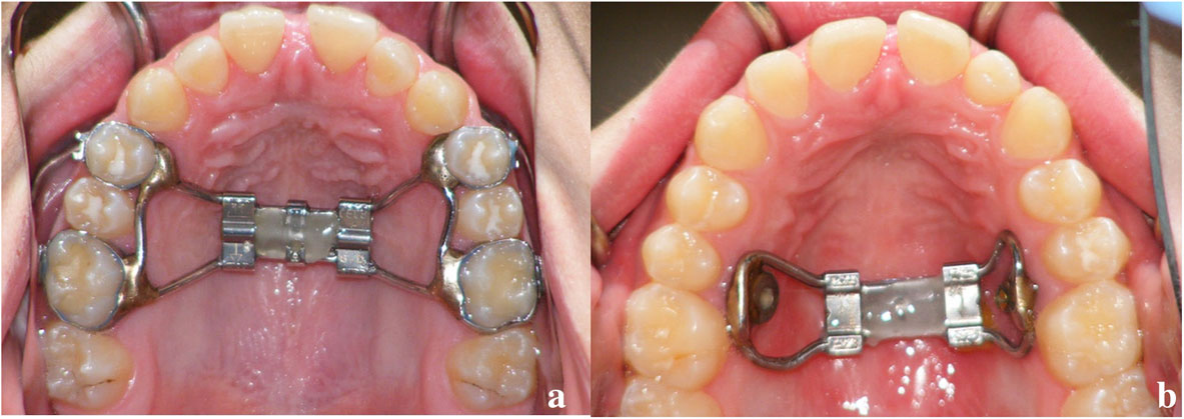
Tooth-borne (**a**) and Bone-borne (**b**) palatal expander used in this study. See the interincisal diastema after maxillary expansion

The characteristics of the RME protocol used in this study have been previously described [22, 23]. Briefly, in the TB group, 20 subjects **(12 female and 8 male, with a mean age of 14.27 ± 1.36 years)** received the **Hyrax-type** expander with bands on the permanent first molars and first premolars (Fig. 1a). In the BB group, **20 subjects (15 female and 5 male, with a mean age of 14.62 ± 1.45 years)** received two miniscrews in the palate between the permanent first molar and the second premolar (length: 12 mm; diameter: 1.5 mm; Straumann GBR System, Andover, MA) that were connected with the expander (Palex II Extra-Mini Expander, Summit Orthodontic Services, Munroe Falls, OH) (Fig. 1b). In both groups, the activation rate of the jackscrew was 0.25 mm/turn and the RME protocol included two turns per day, for a total of 0.5 mm/d. **Clinical assessment of successful expansion protocol was achieved by visual inspection of the opening of the interincisal diastema.** Activations were stopped once overexpansion was achieved, i.e., when the mesiopalatal cusps of the maxillary first permanent molars were in contact with the buccal cusps of the mandibular first permanent molars. **At the end of the activation period, the expansion screw was blocked with flowable composite and the appliance was kept in place for 6 months as retention; during this period, patients did not receive any other type of orthodontic treatment.**

Cone beam computed tomography (CBCT) was performed on all subjects before the expansion (T1) and after the 6 months of retention (T2). **Patients were scanned with the same iCAT CBCT Next Generation Unit (Imaging Sciences International, Hartfield, PA).** The setting protocol included 0.3 voxel, 8.9 seconds, **large field of view (17 x 23 cm)** at 120 kV and 20 mA. The distance between 2 slices was 0.3 mm which provided accuracy in anatomic registration. The protocols used in this study for segmentation, model rendering and deviation analysis were previously validated and clearly described [18, 24].

**Fig. 2:**
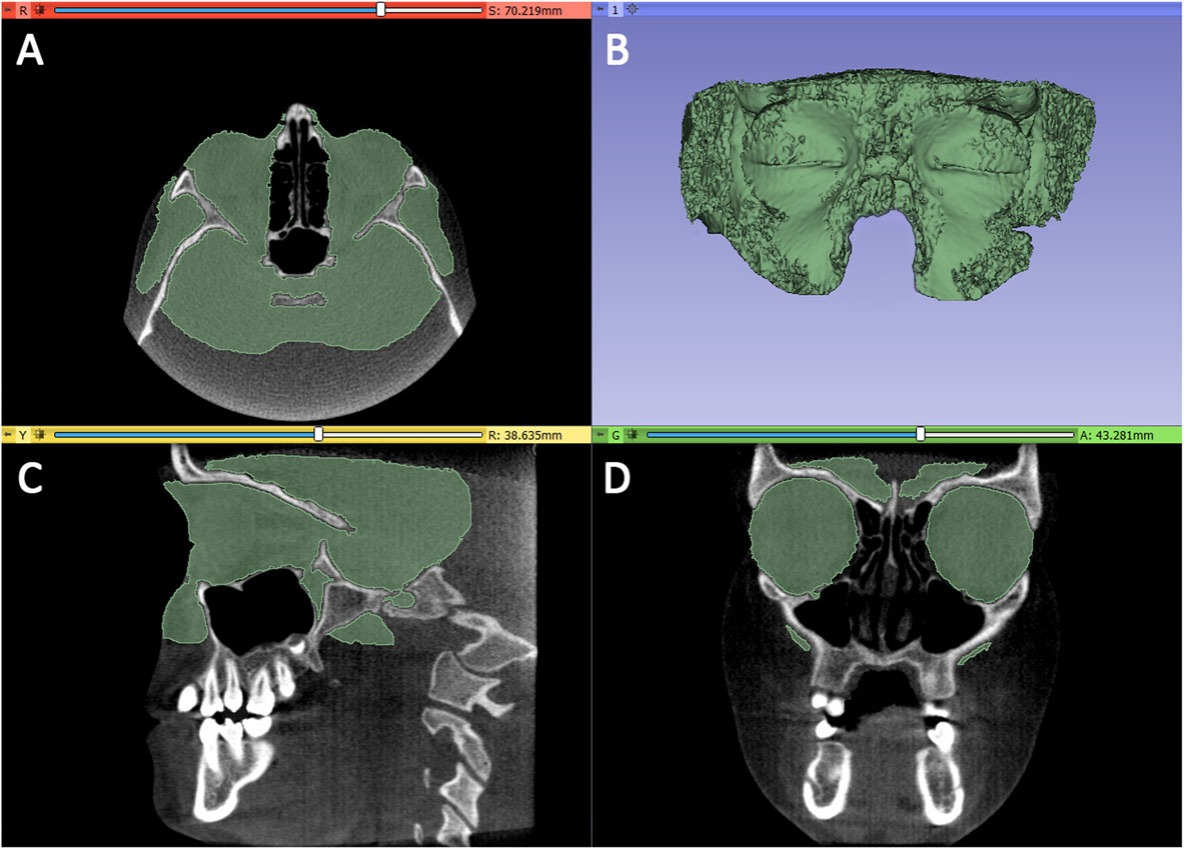
Segmentation mask of soft tissues from CBCT using Slicer 3D software. The ‘fast marching’ algorithm allowed the initial marked regions to propagate toward regions with similar gray levels intensity, excluding bone structures as well as cavities full of air

Step 1- *Generating the preliminary segmentation masks.* The first step was performed by using Slicer 3D software (http://www.slicer.org), to develop the segmentation masks of soft tissues from CBCT. The 'fast marching' algorithm [25] was used to accurately segment the mask of the orbital cavity, in particular this algorithm allowed the initial marked regions to propagate toward regions with similar gray levels intensity, excluding bone structures as well as cavities full of air (Fig. 2). Afterwards, the soft tissue segmentation mask was rendered into a 3D surface models **(*Standard Triangulation Language*** **- .stl).**

**Fig. 3:**
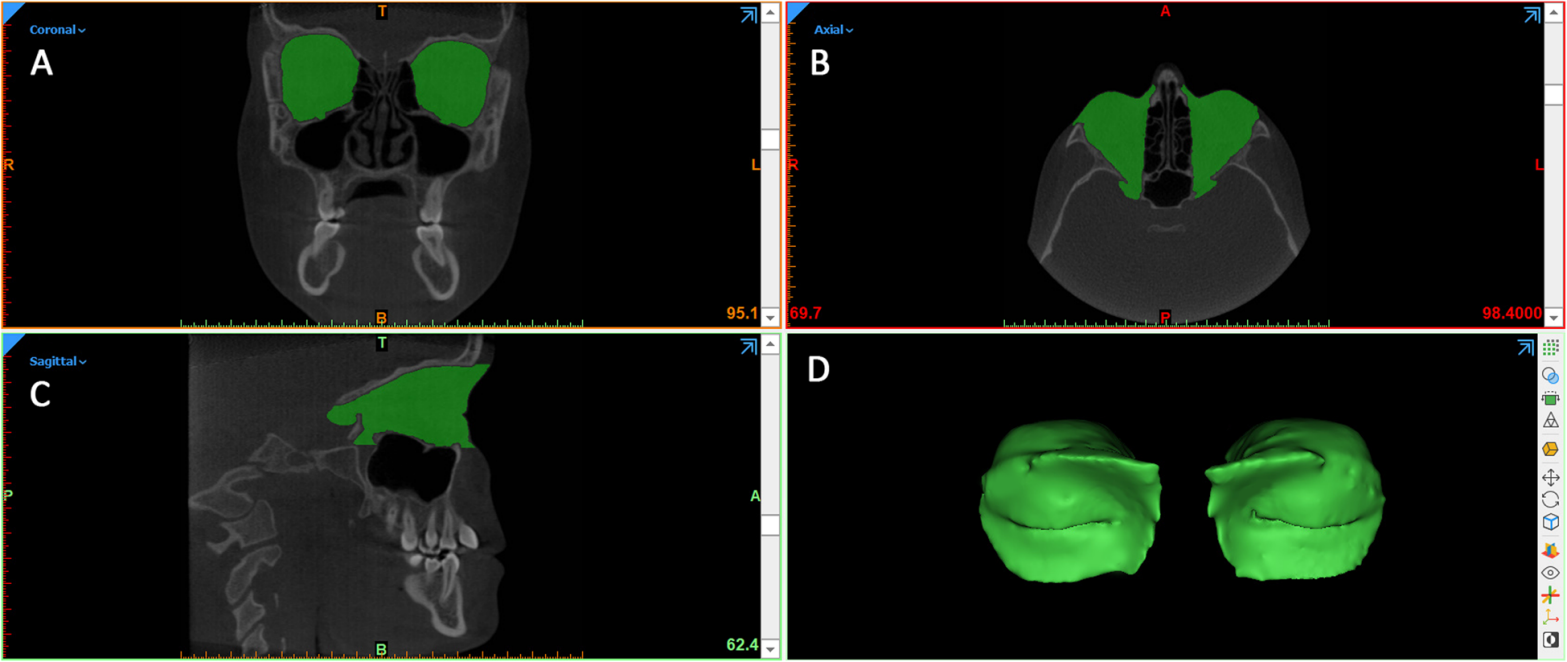
Once imported into Mimics Research software, the soft tissues mask was cleaned up to keep the soft tissues of the orbital region (removal of outside regions)

Step 2 - *Removal of outside regions and cropping of T1 - orbit 3D models*. The 3D model of orbital soft tissue and outside regions and the CBCT scans were imported onto Mimics Research software (vr. 21.0.0.406, Materialise NV, Liege, Belgium) with the same coordinates system (Fig. 3). A new segmentation mask was obtained from the original .stl file and the 'edit mask' function was used to cleaned up the orbital cavity (removal of outside regions). To improve quality and contour delineation, the mask was smoothed and finely adjusted by using the interactive ‘Contour Edit’ function. Afterwards, the edited mask was **converted and rendered** to a 3D model.

**Fig. 4:**
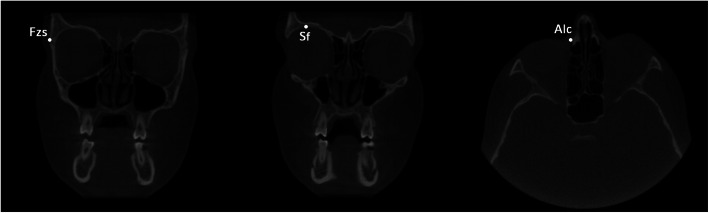
The anterior plane was constructed by selecting two points on the coronal view, i.e., the fronto-zygomatic suture at the external rim of the orbit (FZS) and supraorbital foramen (SF) and by selecting the anterior lacrimal crest (ALC) point on the axial view

**Fig. 5:**
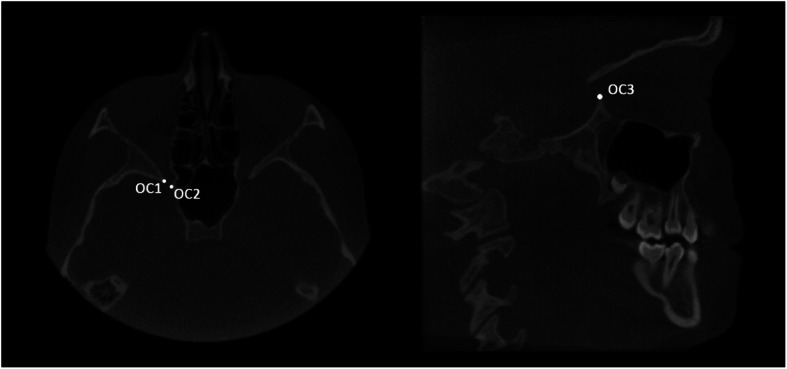
The posterior plane was constructed by selecting the lateral (OC1) and medial (OC2) walls of the optic foramen taken at the most caudal axial view and the most cranial point of the lateral walls (OC3) of the optic foramen taken on the sagittal view

Lastly, two planes cut were selected in order to accurately and reliably define anterior and posterior boundaries of the orbital cavity (Figs. 4, 5). Anterior plane was constructed by selecting two points on the coronal view, respectively the fronto-zygomatic suture at the external rim of the orbit (FZS) and the supraorbital foramen (SF), and the anterior lacrimal crest (ALC) on the axial view. Posterior plane was constructed by selecting two points on the axial view, respectively the lateral (OC1) and medial (OC2) walls of the optic foramen taken at the most caudal scan, and the most cranial point of the lateral walls (OC3) of the optic foramen on the sagittal view.

**Fig. 6:**
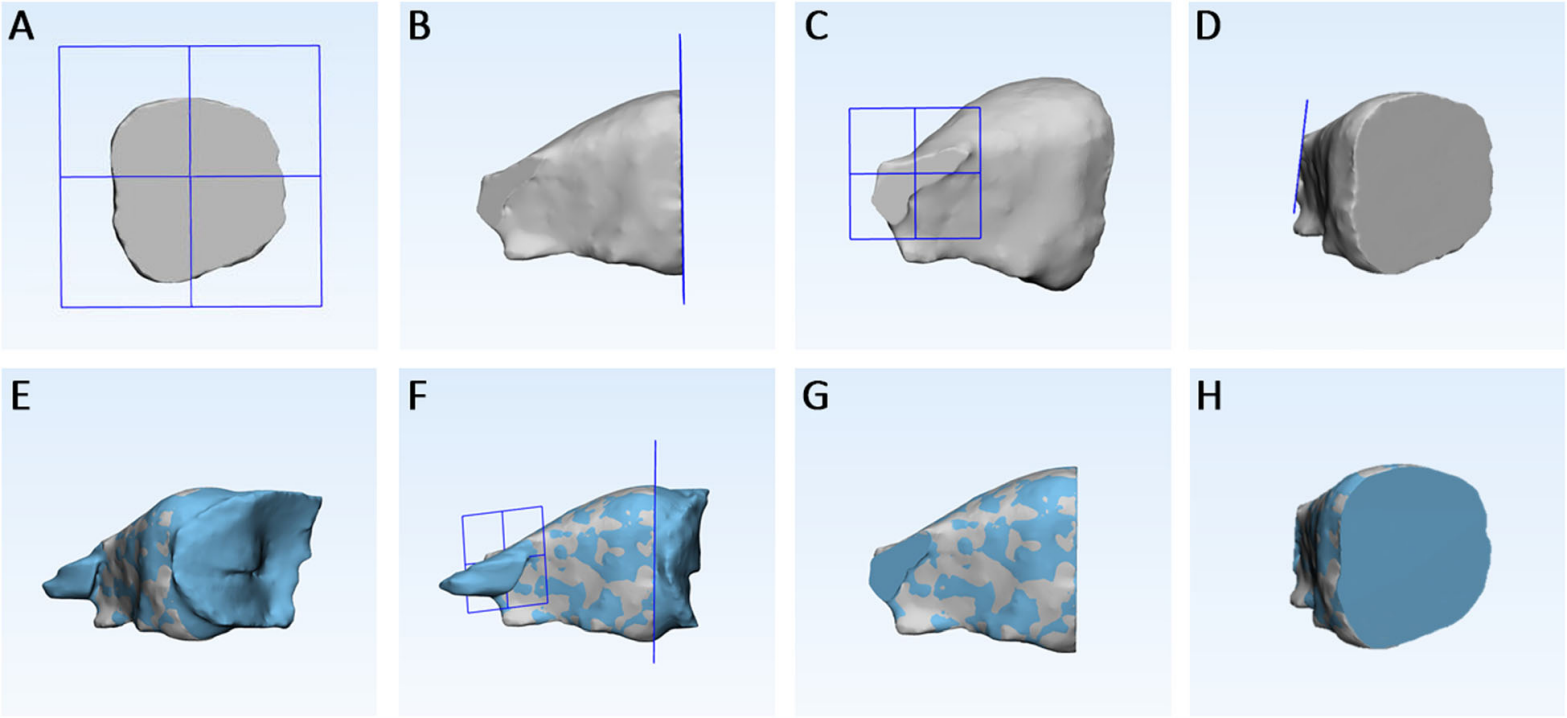
The 3D model of each orbit (T1 and T2) were imported onto 3-matic Medical software. Construction of two cutting planes, one on the anterior surface of T1- orbit 3D model (**a**, **b**) and one on the posterior surface (**c**, **d**) (this planes replicated the planes cut of the segmentation masks); (**e**) surface-based registration between T1 and T2 3D models of orbital cavity, using T1 model as fixed entity; (**f**-**h**) the cut planes previously selected were used to cut the T2 orbit model

**Fig. 7:**
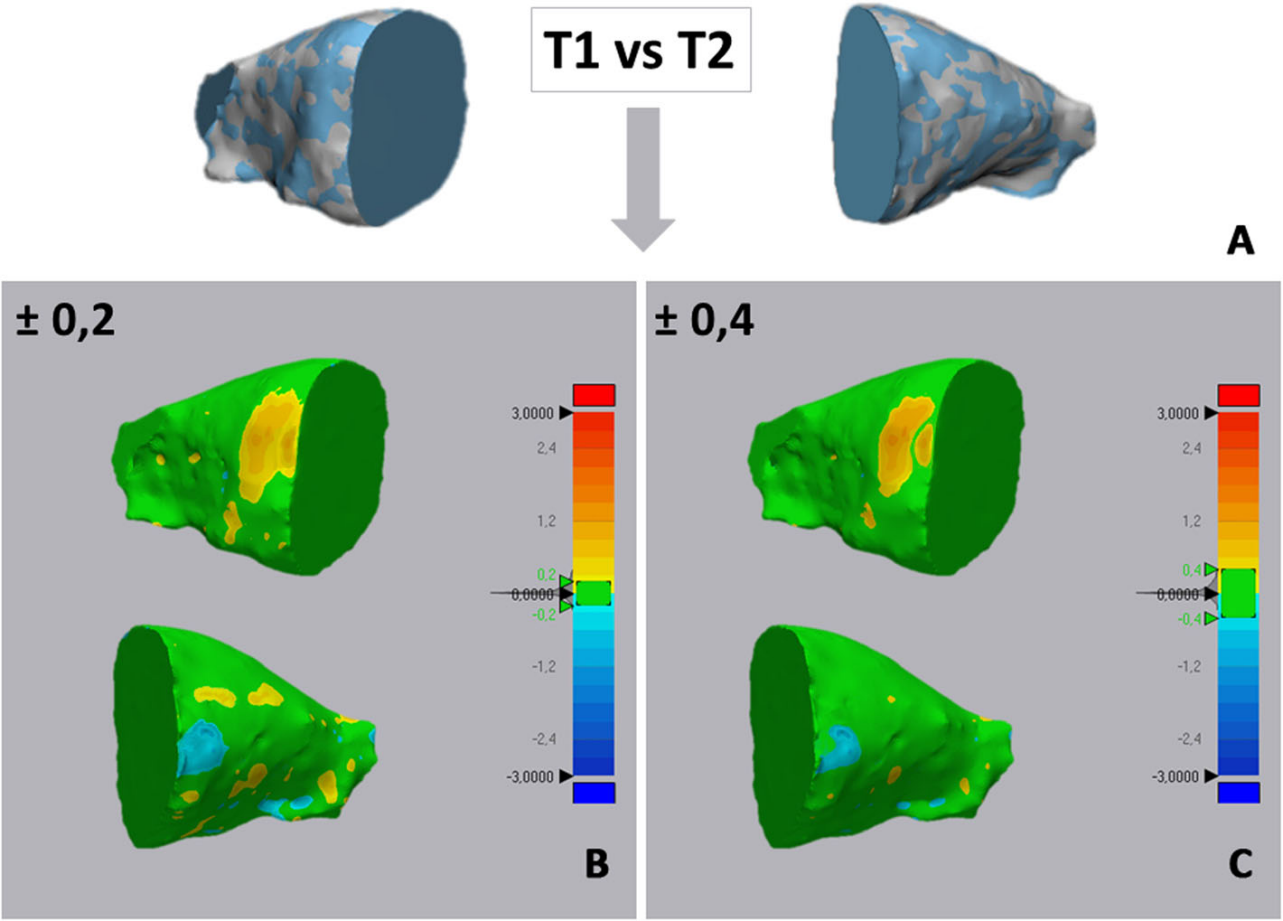
Example of deviation analysis between the T1 and T2 orbital cavity obtained in both tooth-borne and bone-borne expander groups. The colored map shows the deviations (negative blue, positive red) between the mesh models. **a** models superimposition, **b** deviations analysis set at 0.2 mm of tolerance; **c** deviations analysis set at 0.4 mm of tolerance

Step 3 – *Superimposition and cropping of T2 - orbit 3D models.* The 3D model of each orbit (T1 and T2) were imported onto 3-matic Medical software (vr. 13.0.0.188, Materialise NV, Liege, Belgium) with the same coordinates system. Firstly, three points were randomly selected on the anterior and posterior surfaces of T1-3D model, in order to replicate the same planes cut of the segmentation masks (Fig. 6a-d). Using a specific function of the software (‘global registration’), the T1 and T2 3D models of orbital cavity were superimposed using a surface-based registration, with the former used as fixed entity (Fig. 6e). The software automatically calculates the best fitting between the two models. Finally, the cut planes previously selected were used to cut the T2 orbit model (Fig. 6f-h). *Step 4 - 3D Deviation analysis*. After surface-based superimposition, the registered T1 and T2 models of the orbital cavity underwent deviation analysis by using the Geomagic Control X software (version 2017.0.0, 3D Systems, Santa Clara, CA 95054, USA). The analysis was complemented by visualization of the 3D color-coded maps set with a range of tolerance of 0.20 mm and 0.40 mm (Fig. 7). After the deviation analysis, percentages of all the distance values within the tolerance range were calculated.

*Step 5 - 3D Matching percentage calculation and volumetric assessment.* Once the deviation analysis was carried out, the percentages (%) of all the distance values within the tolerance ranges were calculated. These values represented the degree of matching between the pairs of 3D orbital cavity models (T1 and T2).

**The amount of skeletal maxillary expansion was also recorded in each group by calculating the maxillary palatal width. This measure connected the junctions of the hard palate and lingual alveolar bone on the coronal section passing through the maxillary right first molar furcation** [22]**.**

The entire digital **workflow** was performed by a single expert examiner (A.L.G.) who processed only 2 CBCT scans each day to avoid fatigue. All images were standardized for brightness and contrast, and magnified by the same amount. Since post-treatment CBCTs were acquired after removal of the palatal expander, it was possible to blind the examiner regarding the type of appliance used. CBCT records of 10 subjects were randomly selected 4 weeks after the last examination to calculate the intra-observer and **inter-observer variability** and the method error, assuring that both examiners (A.L.G. and L.R.) were not aware of data of the first measurements.

### Statistics

The Shapiro-Wilk Normality Test was preliminarily performed to test the normality of the data. Since the data showed homogeneous variance, parametric tests were used to evaluate and compare measurements. In this respect, the paired Student’s t-test was used to compare T1 and T2 volumetric measurements of the orbital cavity in both TB and BB groups. The unpaired Student’s t test was used to assess if the post-treatment volumetric changes and the percentage of matching between the pre and post-treatment orbit models significantly differed between the two groups. The unpaired Student’s t test was also used to assess if the post-treatment volumetric changes (T1-T2) of the orbital cavity were different between right and left side, in both TB and BB groups. Since no differences were detected (p > 0.05), volumetric data of both sides were merged, thus the final study sample included 40 orbital 3D models in each group, increasing the robustness of the study.

**Linear regression was performed to investigate a cause-effect relationship between skeletal expansion (independent variable) and orbital volume changes (dependent variable). Intra-examiner and inter-examiner** reliability were assessed using intraclass correlation coefficient (ICC) while assessment of the method error was performed using Dahlberg’s formula [26]. Data sets were analyzed using SPSS® version 24 Statistics software (IBM Corporation, 1 New Orchard Road, Armonk, New York, USA).

## Results

Pre and post-treatment volumetric measurements of the orbital cavity are reported in Table 1 along with inferential statistics. In both groups, there was a slight increment of the orbital volume (TB = 0,64 cm^3^ ± 0,07; BB = 0,77 cm^3^ ± 0,22) that was statistically significant (p < 0.0001) (Table 2). Such volumetric increment was statistically significant were between TB and BB groups, with mean difference value of 0, 13 cm^3^ (p < 0.05). **Considering that the sample size included 40 orbital cavities for each group and the significance level set at p < 0.05, the present investigation was adequately powered (94.5 %) to distinguish a mean difference of 0.13 cm**^**3**^ **in the post-treatment volumetric changes of the orbital cavities between TB and BB groups.**

**Table 1:** Data of orbital cavity volumes recorded in both study groups (data included right/left side) and comparison of volumetric changes in orbital cavity between groups

	TB Group					BB Group									
**Sample**	**Orbital volume T1 (cm3)**					**SD**					**Orbital volume T2 (cm3)**	**SD**	**Significance**	**Orbital volume T1 (cm3)**	**SD**
40	24,04	1,71	24,68	1,74	p < 0.0001	23,65	1,58	24,41	1,66	p < 0.0001	0,64	0,07	0,77	0,22	p < 0.05

T1 = before maxillary expansion, T2 = 6 months after maxillary expansion. TB = Tooth-borne, BB = Bone-borne

*P* values are reported according to the paired Student’s t Test for intra-group assessment and according to the unpaired Student’s t Test for comparison of mean differences between groups

*NS* not significant

**Table 2:** Comparison of matching percentage of pre and post-treatment orbital 3D models between tooth-borne (TB) and bone-borne (BB) group

		TB Group		BB group		
	Sample size	Matching	SD	Matching	SD	Significance
		Percentage		Percentage		
		(T1-T2)		(T1-T2)		
Tolerance A	40	83,84%	3,67%	81,87%	2,70%	p > 0.05
Tolerance B	40	94,46%	2,97%	92,89%	2,65%	p > 0.05

T1 = before maxillary expansion, T2 = 6 months after maxillary expansion. *P* values are reported according to the unpaired Student’s t Test

Tolerance A = 0,20 mm; Tolerance B = 0,40 mm. SD = standard deviation

No differences were found in the percentage of matching of T1/T2 orbital 3D models between TB and BB groups (p > 0.05), at both tolerance A (TB = 83,84% ± 3,67%; BB = 81,87% ± 2.70%) and tolerance B (TB = 94,46% ± 2,97%; BB = 92,89% ± 2.65%) (Table 3).

**Table 3:** Linear regression tests model using maxillary expansion as indipendent variable (predictor) and orbital volume as dependent variable

Groups	Maxillary expansion (mm)	Orbital volume (cm3)	Dependent Variables	Predictor Variables	R	R squared	Coefficients		95% Interval Coefficient (B)	
							**Beta**	**Standard error**	**Lower Limit**	**Upper Limit**
TB	1.83 ⏈ 0.42	0.64	Maxillaryexpansion (mm)	Orbital volume (cm3)	0.551	0.319	0.551	0.087	0.061	0.454
BB	2.2 ⏈ 0.33	0.77			0.582	0.325	0.582	0.136	0.088	0.635

Additionally, the colour map analysis of the superimposition of the T1-T2 orbital shells showed red tone (positive mismatching) in a small area located in the anterior upper lateral side of the orbital surface model. Blue tone (negative mismatching) was shown in a small area located in the anterior lower median side of the orbital surface (Fig. 6). The remaining areas demonstrated a prevalence of green, which indicates no significant differences.

**The amount of skeletal maxillary expansion recorded was respectively 1.83 mm (± 0.42) in the TB group and 2.2 mm (± 0.33) in the BB group. The post-treatment increment of the orbital volume showed no significant correlations with the amount of skeletal expansion obtained, being the values of R (linear regression) 0.051 in the TB and 0.582 in the BB group respectively (**Table 3**).**

Concerning the reliability of the methodology, no differences were found between the two readings of the orbital volumes with a correlation indexes of **0,933 and 0,894 respectively for intra-operator and inter-operator variability**. According to the Dahlberg’s formula, the random error for the orbital volumetric measurements was 0,63 cm.^3^

## Discussion

Previous studies have deeply investigated the effects of tooth-borne maxillary expansion confirming that its effects are not limited to the maxilla but also involve adjacent structures, in particular the bones delimiting the nasal cavities and the **circummaxillary** sutures [1–9, 22, 27]. Among these, the zygomatic process, the frontozygomatic suture and the frontal process of the maxilla are part of the orbital cavity. In this respect, it has been reported that subjects underwent RME could feel sensation of pressure in the maxillary, nasal, or orbital areas [28, 29]. Two studies [30, 31] respectively reported the case of an adolescent with neurologic and ophthalmic symptoms (e.g. headache and diplopia) related to tooth-borne maxillary expansion, with the assumption of a potential association of RME and pseudotumor cerebri syndrome (PTCS) related manifestations. Particularly, in the latter study [31] the patient showed a protrusion of the optic nerve head and enlarged perioptic subarachnoid space confirming the diagnosis of PTCS, as assessed via magnetic resonance imaging (MRI). However, the interruption of the maxillary expansion protocol led to the complete resolution of clinical symptoms in 1 week. The authors assumed that PTCS may be a potential complication of RME due to an alteration of the cerebral venous circulation with subsequent impaired venous drainage and increased intracranial pressure. However, it must be underlined that these two findings were case reports with the intrinsic limitations in terms of scientific evidence.

A previous CT study [10] assessed the volumetric dimension and the anterior aperture width of the orbital cavity before and after RME. It was found a slight significant increment of the orbital volume as well as of the anterior width, however authors concluded that such modifications, despite statistically significant, **were not so considerable as to affect the architecture of the orbits or the craniofacial anatomy.**

In the present study, we investigated the volumetric and morphological changes of the orbital cavity after tooth-borne and bone-borne RME basing on the assumption that skeletal anchorage, being more effective in obtaining skeletal improvement of maxillary transversal deficiency, could also induce greater side-effects. To the best of our knowledge, this is the first study in literature for this purpose. We refer to a specific 3D imaging technology involving the fast marching algorithm procedure that allowed the 3D rendering of complex structures [25] such as the orbital cavity.

According to our findings, both TB and BB groups showed a slight increment of the orbital volume at T2 (respectively 0,64 cm^3^ and 0,77 cm^3^) that was statistically significant; this is in agreement with previous findings showing that the orbital volume increased of 0.72 cm^3^ after treatment with maxillary expander. Due to the absence of a control group, these findings should be taken with some caution since part of this volumetric increment could be attributed to the potential residual grow of this region that reach almost 90% at the age of almost 17 years [32, 33] (slight above the mean age of the subjects included in this study). **Despite we accepted the null hypothesis of the study since the volumetric increment of the orbital cavity was statistically significant greater in the BB group, it remains questionable if the mean difference of post-treatment orbital changes between the two groups, i.e. 0,13 cm**^**3**^**, is of clinical relevance. In this regard, we are conscious that our study provides some new evidence as well as new unanswered questions and further studies are certainly required.**

The volumetric assessment of the orbits provides a quantitative evaluation of the potential orbital modification. In order to achieve a qualitative evaluation of these changes and to assess the morphological modification of the orbits, we used the deviation surface analysis between pre and post treatment 3D rendered orbital models. Both TB and BB groups showed a percentage of matching higher than 80% at 0.2 mm of tolerance and higher than 90% at 0.4 mm of tolerance which suggest minimal changes in the shape of the orbital cavity. Interestingly, in both groups, the color-coded map showed a small area of increment (red-to-yellow tones) in the proximity of the inner limit of the fronto-zygomatic suture and a small area of reduction in the proximity of the lateral aspect of the fronto-maxillary sutures. The remaining areas demonstrated a prevalence of green, which indicates no significant differences or potential minimal differences that were within the ranges of tolerance.

Previous evidence [1, 2, 16, 27–29] confirmed the wedge shape opening of the maxilla in the frontal plane with the fulcrum of the rotation approximately located at the frontomaxillary suture. Basing on these findings, it could be postulated that the small areas of morphological modification of the orbits detected in the present study are the consequence of: 1) a lateral stretching of the inner part of the fronto-zygomatic suture, b) a lateral stretching of the fronto-maxillary suture. **These changes are likely the consequence of the stress induced by the orthopedic forces accumulated during the expansion period and a potential relapse during retention period cannot be excluded**. In this respect, the subjects included in the present study were all in the adolescence stage and previous studies confirmed that at this stage there is an increased rigidity of the facial skeleton and of the zygomaticotemporal, zygomaticofrontal, and zygomaticomaxillary sutures that can represent the primary anatomic sites of resistance to RME [34, 35].

Finally, considering the potential effects of rapid maxillary expansion at level of circummaxillary structures, we postulated that **the amount of skeletal expansion obtained with both TB and BB expander could influence the post-treatment increment of the orbital volume found in the present study. However, we found no significant correlations between skeletal expansion and orbital volume changes. This finding could be explained considering that the amount of skeletal maxillary expansion and the orbital volume changes were notably homogeneous among individuals, as showed by the small values of standard deviation, and the limited study sample may have not allowed to detect a causative relationship between the two variables.**

### Limitations

**Data from the BB group of the present study are limited to a specific skeletal anchorage system with two miniscrews placed in the palate between the permanent first molar and the second premolar. Since fulcrum position may vary depending on the design of the expander, further studies including different skeletal anchorage systems, for example the maxillary skeletal expander (MSE)** [36, 37], **are required to evaluate potential side-effects of bone-borne RME on the craniofacial components.**

**In the present study, CBCT examinations were acquired after 6 month of appliance retention, limiting our observations to a short-term period. In this regard, studies with longer follow-up could be required along with a multidisciplinary approach involving ophtalmologists, for evaluating the potential clinical relevance of the findings.**

## Conclusion

According to the results of the present study:

- tooth-borne (TB) and bone-borne (BB) rapid maxillary expansion caused a slight increase of the orbital volume
- this volumetric increment was significantly greater in the BB group compared to the TB group, thus the null hypothesis of the study was accepted
- the area most involved by post-treatment changes were that close to the inner limit of the fronto-zygomatic suture and that close to the lateral aspect of the fronto-maxillary sutures
- it is questionable if the increment of orbital volume and the morphological changes detected in the present study are of relevance from a clinical perspective.

## Data Availability

The datasets used and/or analyzed during the current study are available from the corresponding author on reasonable request.
